# Effect of asthma, COPD, and ACO on COVID-19: A systematic review and meta-analysis

**DOI:** 10.1371/journal.pone.0276774

**Published:** 2022-11-01

**Authors:** Yuka Uruma, Toshie Manabe, Yuji Fujikura, Motoyasu Iikura, Masayuki Hojo, Koichiro Kudo

**Affiliations:** 1 Nagoya City University Medical School, Aichi, Japan; 2 Nagoya City University Graduate School of Medical Sciences, Aichi, Japan; 3 Nagoya City University West Medical Center, Aichi, Japan; 4 Division of Infectious Diseases and Respiratory Medicine, Department of Internal Medicine, National Defense Medical College, Saitama, Japan; 5 Department of Medical Risk Management and Infection Control, National Defense Medical College Hospital, Tokorozawa, Japan; 6 Department of Respiratory Medicine, National Center for Global Health and Medicine, Tokyo, Japan; 7 Yurin Hospital, Tokyo, Japan; 8 Waseda University, Institute for Asia Human Community, Tokyo, Japan; Kyung Hee University School of Medicine, REPUBLIC OF KOREA

## Abstract

**Introduction:**

The prevalence of asthma, chronic obstructive pulmonary disease (COPD), and asthma-COPD overlap (ACO) in patients with COVID-19 varies, as well as their risks of mortality. The present study aimed to assess the prevalence of asthma, COPD, and ACO as comorbidities, and to determine their risks of mortality in patients with COVID-19 using a systematic review and meta-analysis.

**Methods:**

We systematically reviewed clinical studies that reported the comorbidities of asthma, COPD, and ACO in patients with COVID-19. We searched various databases including PubMed (from inception to 27 September 2021) for eligible studies written in English. A meta-analysis was performed using the random-effect model for measuring the prevalence of asthma, COPD, and ACO as comorbidities, and the mortality risk of asthma, COPD, and ACO in patients with COVID-19 was estimated. A stratified analysis was conducted according to country.

**Results:**

One hundred one studies were eligible, and 1,229,434 patients with COVID-19 were identified. Among them, the estimated prevalence of asthma, COPD, and ACO using a meta-analysis was 10.04% (95% confidence interval [CI], 8.79–11.30), 8.18% (95% CI, 7.01–9.35), and 3.70% (95% CI, 2.40–5.00), respectively. The odds ratio for mortality of pre-existing asthma in COVID-19 patients was 0.89 (95% CI, 0.55–1.4; *p* = 0.630), while that in pre-existing COPD in COVID-19 patients was 3.79 (95% CI, 2.74–5.24; *p*<0.001). France showed the highest prevalence of asthma followed by the UK, while that of COPD was highest in the Netherlands followed by India.

**Conclusion:**

Pre-existing asthma and COPD are associated with the incidence of COVID-19. Having COPD significantly increases the risk of mortality in patients with COVID-19. These differences appear to be influenced by the difference of locations of disease pathophysiology and by the daily diagnosis and treatment policy of each country.

## Introduction

Severe acute respiratory syndrome coronavirus 2 (SARS-CoV-2) was first detected in Wuhan, China [1], and it is the causative agent of the coronavirus disease 2019 (COVID-19) pandemic [2]. As many as 50% of patients have reported having at least one comorbidity with COVID-19 [3]. Among them, the highest prevalent comorbidity was hypertension (21.1%), followed by diabetes (9.7%), cardiovascular disease (8.4%), and respiratory system disease (1.5%) [3]. However, the prevalence of asthma, as a comorbidity of patients with COVID-19, has been reported to vary from 1.10% [4] to 36.3% [5]. Additionally, the prevalence of chronic obstructive pulmonary disease (COPD) in COVID-19 ranges from 0.70% [6] to 70.60% [7] and that of asthma-COPD overlap (ACO) ranges from 0.40% [8] to 29.40% [7]. Previous reports have indicated that the global prevalence of asthma in adults is estimated to be 4.3% [9], that of COPD is estimated to be 12.16% [10], and that of ACO ranges from 0.9% to 11.1% [11]. While some studies have reported that asthma, COPD, and ACO are related to an increase in the mortality rate of COVID-19 [12, 13], some studies have reported that they may not be risk factors or may not increase the mortality of COVID-19 [14–17]. However, studies on detailed examinations of the prevalence and risk of mortality of asthma, COPD, and ACO in patients with COVID-19 are still lacking.

Therefore, this study aimed to systematically review and integrate the data from studies with various results on the prevalence of asthma, COPD, and ACO in patients with COVID-19. We also aimed to determine the mortality risks of asthma, COPD, and ACO in patients with COVID-19.

## Methods

This systematic review and meta-analysis was conducted in accordance with the Preferred Reporting Items for Systematic Review and Meta-Analysis (PRISMA) statement and the statement by the Meta-analysis of Observational Studies in Epidemiology (MOOSE) group [18–20].

### Search strategy

Two investigators (Y.U. and T.M.) independently searched for eligible studies in PubMed, the Cochrane Library, and MedRxiv from inception to 27 September 2021. We used the following key words: “asthma” OR “asthmatic” OR “COPD” OR “Chronic Obstructive Lung” OR “Chronic Obstructive Pulmonary Disease” OR “chronic bronchitis” OR “pulmonary emphysema” OR “pulmonary disease” OR “Chronic Obstructive” OR “Chronic Obstructive Airway Disease” OR “COAD” OR “Chronic Obstructive Lung Disease” OR “Chronic Airflow Obstruction” OR “Obstructive Lung Disease” OR “Obstructive pulmonary Disease” OR “Lung Disease” OR “ACO” OR “asthma-COPD overlap” OR “Asthma-chronic obstructive pulmonary disease overlap syndrome” OR “Asthma and chronic obstructive pulmonary disease overlap syndrome” OR “asthma-COPD overlap syndrome” OR “asthma-COPD” OR “ACOS” OR “mixed asthma-COPD phenotype” OR “Asthma combined with COPD” OR “coexistence of asthma and COPD” OR “coexistence of asthma and COPD” OR “COPD with asthmatic features” OR “overlap of asthma-COPD” AND “COVID-19” OR “novel coronavirus” OR “new coronavirus” OR “emerging coronavirus” OR “2019-nCoV” OR “SARS-CoV-2” OR “COVID” OR “coronavirus” OR “nCov” OR “coronavirus disease 2019” OR “coronavirus 2019”. We also reviewed the reference lists of eligible studies using Google Scholar and performed a manual search to ensure that all appropriate studies were included.

### Eligibility criteria and outcome measures

Studies fulfilling the following selection criteria were included in the meta-analysis: (1) randomized, clinical trials, observational studies, and case series involving >20 patients written in English; and (2) patients with positive laboratory-confirmed SARS-CoV-2 infection who had asthma, COPD, or ACO as comorbidities. The exclusion criteria were as follows: (1) systematic reviews, (2) reviews, (3) animal experimental reports, (4) ≤20 patients in case series, (5) insufficient or incomplete data, (6) unpublished articles, and (7) pediatrics reports.

### Data extraction

Two reviewers (Y.U. and T.M.) extracted the data independently. Articles that were retrieved in the search were stored in a citation manager. After removing redundant articles, titles, and abstracts, full-text articles were then investigated. We extracted the following data: study design, observational period, study site, and inclusion/exclusion criteria of each study. Outcome variables were extracted into predesigned data collection forms. We verified the accuracy of the data by comparing the collection of each investigator, and any discrepancies were resolved through discussion.

### Level of evidence

The level of evidence was determined using the Grading of Recommendations, Assessment, Development, and Evaluations (GRADE) framework, which classifies the level of evidence for each outcome on the basis of the risk of bias, imprecision, inconsistency, indirectness, and publication bias [21]. The authors classified the evidence level for each eligible study in accordance with the revised grading system for recommendation in the evidence-based guideline [22] (S1 Table).

#### Data analysis

In the meta-analysis, we estimated the odds ratios (ORs) or the proportions of patients for primary outcome variables with 95% confidence intervals (CIs) using the random-effects model (generic inverse variance method). To assess the proportions of the outcome variables in patients with COVID-19, the standard error was calculated using the Agresti-Coull method [23]. Heterogeneity among the original studies was evaluated using the *I*^2^ statistic [24]. Publication bias was examined using a funnel plot. For all analyses, significant levels were two-tailed, and *p*<0.01 was considered significant. All statistical tests were performed using Review Manager (RevMan) ver. 5.4.1 (Cochrane Collaboration, Copenhagen, Denmark) [25].

#### Ethics approval and consent to participate

The institutional review board and patient consent were not required because of the review nature of this study.

## Results

### Study selection and characteristics

**Fig 1:**
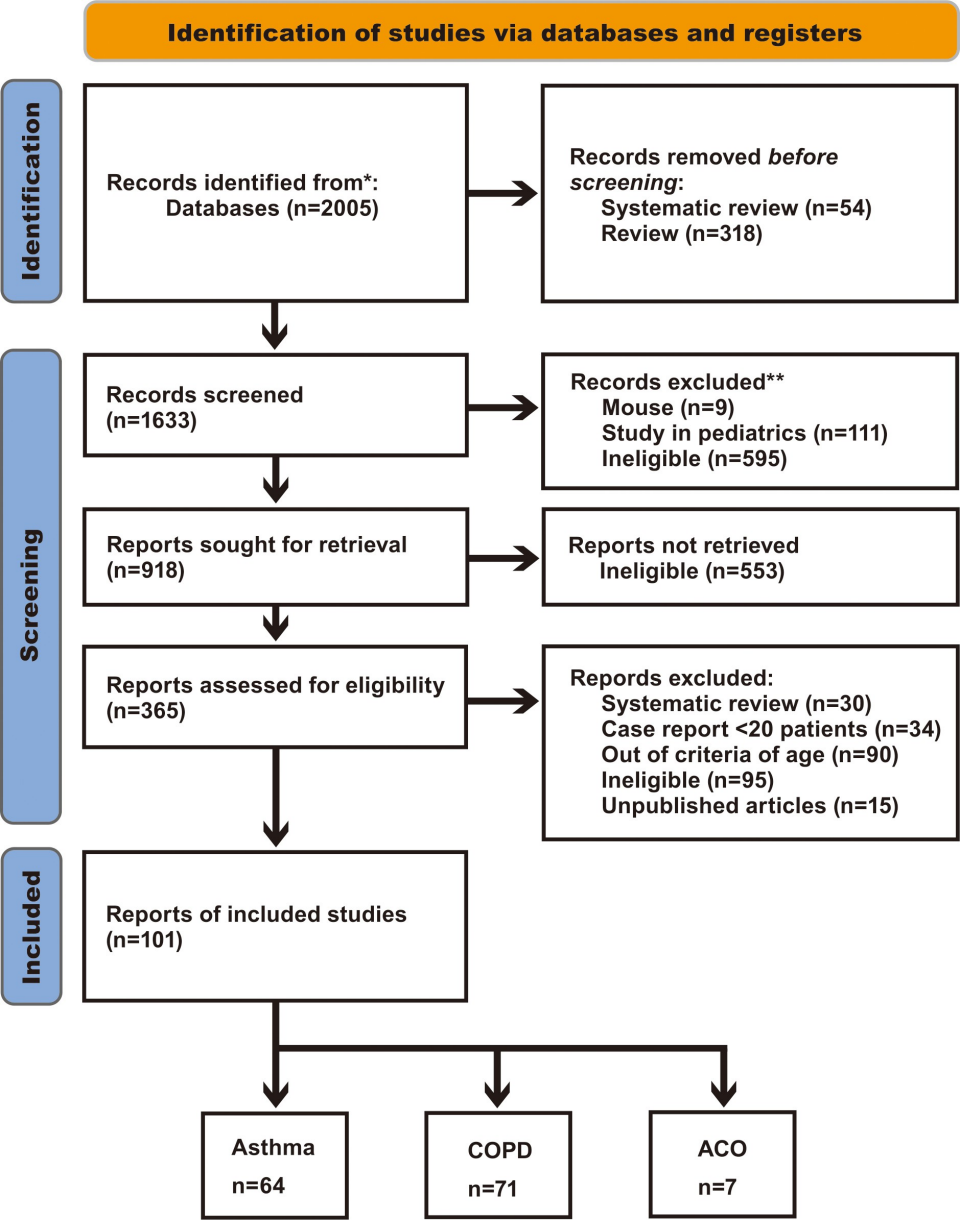
PRISMA flow diagram. N indicates the number of articles.

Of the 2005 references screened, 101 studies reported the outcome variables (Fig 1).

We analyzed 64 studies on asthma, 71 on COPD, and 7 on ACO. Thirty-three studies were duplicated for asthma and COPD, two for asthma and ACO, and four for asthma, COPD, and ACO. Table 1 shows the characteristics of the included studies.

**Table 1:** The characteristics of the included studies.

Study, year	Country	Observational period	Study Design	No. of participants	Sex-Male, n (%)	Age, Median (IQR) or mean±SD, years	Severity of COVID-19, n (%)	Standard of the evidence level
Zhang JJ, 2020 [26]	China	Jan.16-Feb.3, 2020	-	140	71 (50.7)	57 (range, 25–87)	Nonsevere 82 Severe 58	2-
Wang L, 2020 [27]	China	Jan.1-Feb.6, 2020	Retrospective single-center study	339	166	69 (65–76)	Moderate100(29.5) severe159(46.9) critical80(23.6)	2-
Bhatraju PK, 2020 [28]	USA	Feb.24-Mar.9, 2020	-	24	15 (63)	64±18		2-
Turan O, 2021 [29]	Turkey	Mar-Aug, 2020	Multicenter, retrospective cohort study	1069	634	>18		2-
Barrasa H, 2020 [30]	Spain	Mar.4-Mar.31, 2020	-	48	27 (56)	63 (12)≥18		2-
Mahdavinia M, 2020 [31]	USA	Mar.12-Apr.3 2020	-	935	417	≥18		2-
Li P, 2020 [32]	China	Jan.31-Feb.20, 2020	-	204	100	68 (64–75) Range, 60–95	Mild 64.7%, severe 33.3%, critical 2%	2-
Lian J, 2020 [33]	China	Jan.17-Feb.12, 2020	retrospective	136	58	68.28±7.314	Mild 102, severe 22, critical 12	2-
Iaccarino G, 2020 [34]	Italy	Mar.9-Apr.9, 2020	Cross-sectional, multicenter, observational study	1591	64%	66.5±0.4 range18-101		2-
Toussie D, 2020 [35]	USA	Mar.10-Mar.26, 2020	Retrospective study	338	210 (62)	39 (31–45) Between 21 and 50	Low chest radiograph severity score 0–1:202 2–6:136	2-
Argenziano M, 2020 [36]	USA	Mar.1-Apr.5, 2020	Retrospective case series	1000	596	Emergency department55 (40–69) In hospital 64 (51–77) ICU, 62 (52–72)		2-
Cummings MJ, 2020 [37]	USA	Mar.2-Apr.1, 2020	Prospective cohort study	257 critical	171 (67)	62 (51–72)		2-
Zhu Z, 2020 [38]	USA	Mar.16 2020-	Population-based prospective cohort study	641	288 (45)	56±8 Aged 40–69		2-
Grandbastien M, 2020 [39]	France	Mar.4-Apr.6, 2020	Monocentric, retrospective, cohort study	106	66 (62.3)	63. 5(54.2–72.0)		2-
Yao Y, 2020 [40]	China	-Mar.10, 2020	Retrospective, multicenter, cohort study	171	92 (53.8)	50.5±15.2	Severe 71(41.5), critical 29(17.0)	2-
Lieberman-Cribbin W, 2020 [41]	USA	Feb.29-Apr.24, 2020	-	6245	49%	57		2-
Wang J, 2020 [42]	China	Jan.24-Feb.23, 2020	Retrospective study	307	156 (50.8)	57.65±15.754	Mild/moderate 259(84.6) Severe/critical 48(15.6)	2-
Aggarwal A, 2020 [43]	India	Apr.10-Apr.30, 2020	Retrospective, single-center case series	32	19 (59.4)	54. 5 (46.25–60)	Severe 24 Non-severe 8	2-
Caminati M, 2020 [44]	Italy	Mar.1-Apr.30, 2020	-	Brescia20 Verona6	Brescia8 Verona3	Brescia41-77 (mean 61.5) Verona55-79 (69.3)		2-
Bello-Chavolla OY, 2020 [4]	Mexico	-June.3, 2020	Population-based statistics	20804	12257 (58.9)	≥ 60		2-
Bravi F, 2020 [45]	Italy	-Apr.24, 2020	Case-control, retrospective study	1603	47.3%	58.0 (20.9) All adults	Mild957 Severe454 Very severe/lethal192	2-
Song J, 2021 [46]	China	Feb.1-Mar.6 2020	Retrospective observational study	961	500 (52.0)	63 (49–70)	Nonsevere719(74.8) Severe242(25.2)	2-
Wang L, 2020 [47]	USA	Mar .3-June.8, 2020	-	1827	595	54 (37–66)	Hospitalized 565, Non-hospitalized 1262	2-
Canevelli M, 2020 [48]	Italy	Feb.21-Apr.29, 2020	-	2687	1807	Natives 78.3±10.8 Migrants 71.1±13.1		2-
De Vito A, 2020 [49]	Italy	Mar.8-Apr.8, 2020	Retrospective, monocentric study	87	56 (64.4)	72 (62.5–83.5)		2-
Atkins JL, 2020 [50]	UK	Mar.16-Apr.26, 2020	-	507	311	74.3 (4.5) Aged 65 and older		2-
Zhao Z, 2020 [51]	USA	Mar.9-Apr.20, 2020	Retrospective study	641	Died 53 (64.6), ICU admission136 (69.7) General admission222 (55.8)	Died 77 (66–85) ICU admission 60 (50–70) General admission 58 (46–71)		2-
Pérez-Sastré MA, 2020 [52]	Mexico	Feb.28-June.21, 2020	-	159017	(52.2)	≥20		2-
Guner R, 2020 [53]	Turkey	Mar.10-Apr.10, 2020	-	222	132 (59.5)	50.6±16.5 (18–93)	Mild172 Critical50	2-
Somani SS, 2020 [54]	USA	Feb.27-Apr.12	Retrospective cohort study	2864	1663	≥18		2-
Yang JM, 2020 [55]	Korea	Jan.1-May.15, 2020	Propensity-score-matched nationwide cohort	7430	2970 (40.5)	49.0±19.9		2-
Campioli CC, 2020 [56]	USA	Feb.1-May.15, 2020	Retrospective study	251	103 (41.0)	53 (27) adult		2-
He Y, 2020 [57]	China	Jan.20-Apr.1 2020	-	336	201 (59.8)	65 (50–77)	severe	2-
Mushtaq J, 2021 [58]	Italy	Feb.25-Apr.9, 2020	Retrospective single-center study	697	465 (66.7)	62 (52–75)		2-
Goel N, 2020 [59]	India	May.8-July.3, 2020	Retrospective observational study	35	20 (57.1)	46±17	Symptomatic 29(82.9%), asymptomatic 6(17.1%)	2-
Brendish NJ, 2020 [60]	UK	Mar.20-Apr.29, 2020	Prospective, interventional, non-randomised study	352	202 (57.4)	68 (50–80)		2-
Ioannou GN, 2020 [61]	USA	Feb.28-May.14, 2020	Longitudinal cohort study	10131	9221 (91.0)	63.6 (16.2) ≥18		2-
Xiong Q, 2021 [62]	China	-Mar.1, 2020	Longitudinal study	538	245 (45.5)	52.0 (41.0–62.0) From 20 to 80	General 331(61.5), severe 180(33.5), critical 27(5)	2-
Seaton RA, 2020 [63]	UK	Apr.20-30, 2020	-	531	274 (51.6)	72(61–82) Range25-104		2-
Abrams MP, 2020 [64]	USA	Mar.1-Apr.3, 2020	cohort	133	74 (55.6)	81.0 (70.5–88.0)	Arrhythmic death11 Nonarrhythmic death122	2-
Akpinar G, 2021 [65]	Turkey	Mar.1-May.31, 2020	Retrospective cross-sectional design	88	46	48.0±17.3		2-
Schiavone M, 2021 [66]	Italy	Feb.23-Apr.1, 2020	Retrospective study	844	521 (61.7)	63.4±16.1		2-
Calmes D, 2020 [67]	Belgium	Mar.18-Apr.17 2020	-	596	294	≥35		2-
Rial MJ, 2021 [68]	Spain	Mar-June, 2020	Multicenter retrospective cohort	35	14	≥20		2-
Robinson LB, 2020 [69]	USA	Mar.8-Apr.27, 202	Matched cohort study	403	191	≥18		2-
Cates J, 2020 [70]	USA	Mar.1-Mar.31, 2020	-	3948	3710 (94.0)	70 (61–77)		2-
Şanlı DET, 2020 [71]	Turkey	Mar.11-Apr.11, 2020	Local institutional reveiw	102	73 (72)	48.62±14.42 Ranging 19–94		2-
Hussein MH, 2020(USA) [72]	USA	Mar.15-June.9, 2020	Multi-center retrospective study	502	238	Mean age 60.7 ≥18	qSOFA score, CURB65 score	2-
Liao SY, 2021(USA) [5]	USA	Mar.11-June.23, 2020	Prospective observational study	113	53 (47)	50±16		2-
Tabarsi P, 2021 [73]	Iran	?	Randomized controlled trial	84	65	IVIg, 54.29±12.85 Control group, 52.47±14.49 Between 18 and 65	All severe patients	1-
Lee SC, 2020 [74]	Korea	Jan.20-May.27 2020	Retrospective cohort study	7272	2927	≥20	Non-severe, severe	2-
Jiang Y, 2020 [75]	China	Jan.30-Mar.8, 2020	Retrospective observational study	281	143	≥60		2-
Xiao J, 2020 [76]	China	Dec.25, 2019-Feb.16, 2020	Retrospective single-center study	243	105 (43.2)	47.0 (range20-89)	Moderate203, severe/critical 40	2-
Signes-Costa J, 2021 [77]	Spain	Mar.23-May.5, 2020	Retrospective, multicenter, cohort study	5847	3432	65.1±16.6		2-
Bello-Chavolla OY, 2020 [78]	Mexico	Mar.16-Aug.17, 2020	-	3007	Non-severe1227 (50.5) Severe403 (70.1)	Non-severe 44 (33–55) Severe 56 (47–66)	Non-severe2432 Severe 575	2-
Lokken EM, 2020 [79]	USA	Jan.21-Apr.17, 2020	Retrospective study	46	0 Pregnant women	29 (26–34)		2-
Gómez Antúnez M, 2021 [80]	Spain	Mar 2020	Retrospective cohort study	10420	5893 (56.7)	69 (55–79)		2-
Ferastraoaru D, 2021 [8]	USA	Mar.14-Apr.27, 2020	Retrospective study	4558	(31.8)	Asthma 60.5±17.07		2-
Monterrubio-Flores E, 2021 [81]	Mexico	Feb.28-July.31, 2020	-	406966	216908 (53.2)	≥20		2-
Mortaz E, 2021 [82]	Iran	Apr.10-May.9, 2020	retrospective observational study	29	17	54.45±2.536 (range, 32–79)		2-
Değerli E, 2021 [83]	Turkey	Mar.23-Oct.23, 2020	Retrospective study	45	23 (51)	60.3±15.65		2-
Laake JH, 2020 [84]	Norway	Mar.10-June.19, 2020	National cohort	217	162	63 (54.2–72.2)		2-
Lee SC, 2021 [85]	Korea	Jan.20-May.27, 2020	Retrospective cohort study	4610	1710	≥40		2-
Jungo S, 2021 [86]	France	Apr.1-Apr.29, 2020	-	79	37 (46.8)	44 (36–53) Range, 21–86	COVID-19-related phenotypes 68(86.1)	2-
Cao L, 2021 [87]	USA	Mar-Sep, 2020	Prospectively collected cohort	343	192	>18		2-
Fong WCG, 2021 [88]	UK	Mar.1-May.31, 2020	retrospective	6638(with, w/o covid)	3079 (46.4)	65 (42–79)		2-
Jongbloed M, 2021 [89]	Netherland	Feb.28-Apr.1, 2020	Retrospective cohort study	303	195 (64)	72±12		2-
Artero A, 2021 [90]	Spain	Mar.1-May.28, 2020	Multicenter retrospective cohort study	10238	5924 (57.9)	66.6±16.2		2-
Ho KS, 2021 [91]	USA	Mar.7-June.7, 2020	Retrospective multicenter cohort study	10523	5707	58.35±18.81		2-
Yoshida Y, 2021 [92]	USA	Feb.27-July.15, 2020	Retrospective case series	776	365 (47.3)	60.5 (16.1) >18		2-
Nanda S, 2021 [93]	USA	Jan.1-May.23, 2020	retrospective	1169	575 (49.2)	43.9 (17.6) [range18.0–99.0]		2-
De Vito A, 2021 [94]	Italy	Apr.9-May.31, 2020	Observational retrospective cohort study	264	99 (37.5)	81.93±10.11	Symptomatic 132 Asymptomatic 132	2-
Rodriguez C, 2021 [95]	France	Mar.9-30, 2020	-	104	59	Outpatient 50 (range, 19–87) Hospitalized 61 (31–82) ICU, 68 (33–90)		2-
Garibaldi BT, 2021 [96]	USA	Mar.4-Aug.29, 2020	Retrospective comparative effectiveness research	2299	1193 adults	All remdesivir, 60 (46–69) All control, 60 (44–74)		2-
Giovannetti G, 2021 [97]	Italy	May.18-July.25, 2020	Prospective observational study	38	27 (71.1)	60.6 (10.4) Between 18 and 75	Mild11(28.9) Moderate11(28.9) Severe3(7.9)	2-
Khan MS, 2021 [98]	USA	Jan.1-June.15, 2020	Retrospective, observational cohort study	470	224 (47.7)	≥18		2-
Tsai S, 2021 [99]	USA	Feb.24-Nov.25, 2020	Retrospective cohort	8308	0 All women	50.69±12.80 Adult		2-
Lobelo F, 2021 [100]	USA	Mar.3-Oct.29, 2020	Retrospective cohort	5721	2416 (42.2)	44.8 (15.7) ≥18		2-
Chatterjee A, 2021 [101]	Netherland	Mar.1-July.1, 2020	Retrospective study	2337	Non-mortality1078 (60.9) Mortality393 (69.2)	Non-mortality, 65 (55–75) Mortality, 77 (70–83)	Non-mortality1769 Mortality568	2-
Yordanov Y, 2021 [102]	France	Mar.9-Aug.11, 2020	Prospective cohort	7320	2301 (31.5)	43.0±13.9		2-
Riou M, 2021 [103]	France	June-Dec, 2020	descriptive	81	59 (73)	61 (51–68)	Mild-to-moderate 21, severe 15, critical 45	2-
Wei W, 2021 [104]	USA	June.1-Dec.9, 2021	Retrospective study	206741	85228	46.7 (17.8) ≥18	-	2-
Hou X, 2021 [105]	China	Jan.28-Feb.25, 2020	Single-center retrospective cohort study	113	61 (54)	55.1±14.2	Severe113	2-
Valverde-Monge M, 2021 [106]	Spain	Jan.31-Apr.17, 2020	Retrospective analysis	2539	1275	NCRD 61.1±19.3, CRD 71.4±14.8	-	2-
Cosio BG, 2021 [107]	Spain	Mar.15-Apr.30, 2020	Case-control study	52	48 (92.3)	72.96±10.75	-	2-
Adir Y, 2021 [108]	Israel	Mar.1-Dec.7, 2020	Case-control study	8242	4343	43.3±20.4	Moderate, severe	2-
Sen P, 2021 [7]	USA	Mar.8-Sap.16, 2020	-	1288	499 (38.8)	63.7 (15.2)≥35	-	2-
Chandel A, 2021 [6]	USA	Mar.1-June.9, 2020	Multicenter retrospective observational study	272	180	57±13	-	2-
Chaudhary S, 2021 [109]	USA	Mar.15-May.10, 2020	Single-center retrospective observational study	128	71	68 (61–75.5) All adult	-	2-
Williamson EJ, 2020 [110]	UK	Feb.1-May.6, 2020	Cohort study	10926	6126 (0.07)	≥18	-	2-
Abayomi A, 2021 [111]	Nigeria	Feb.27-Jul.6, 2020	Retrospective cohort study	2075	1379	40 (32–50) Range18-98	Mild/asymptomatic 1179, moderate 743, severe 107, critical 42	2-
Liu YH, 2021 [112]	China	Feb.10-Apr.10, 2020	Cross-sectional study	1539	738 (47.95)	69 (66–75)	Severe238 Non-severe1301	2-
Munblit D, 2021 [113]	Russia	Dec.2-Jan.14, 2020	Longitudinal cohort study	1358	675	57 (47–67)	Mild841(61.9) Moderate479(35.3) Severe38(2.8)	2-
Sandoaval M, 2021 [114]	USA	Mar.1-Dec.7, 2020	Retrospective registry-based chart reveiw	1853	704(38.0)	24 (21–27) From 18 to 29	-	2-
Lokken EM, 2021 [115]	USA	Mar.1-June.30, 2020	Multicenter retrospective cohort study	240	0 (pregnant woman)	28 (24–34)	Mild218(90.8) Severe18(7.5) Critical4(1.7)	2-
Cataño-Correa JC, 2021 [116]	Colombia	Mar-Aug, 2020	-	399	235(58.9)	>18	-	2-
Meza D, 2021 [117]	USA	Feb.2021	-	387008	COPD 3949, no COPD 126324	COPD 70.5, no COPD 57.9 Aged over 35	-	2-
Sun Y, 2021 [118]	China	Feb.2-Mar.25, 2020	Retrospective study	268	139(51.9)	57.75 (67–73) Range, 20–88	Severe 96 Non-severe 172	2-
Fernández-Martínez NF, 2021 [119]	Spain	Mar.1-Apr.15, 2020	Observational longitudinal study	968	530(55)	67 (55–77)	-	2-
Cosma S, 2021 [120]	Italy	Sep.20-Jan.9, 2020	Case-control study	21	0	≥18	-	2-
Chudasama YV, 2021 [121]	UK	Mar.16-July.26, 2020	-	1706	981(57.5)	68 (range48-85)	severe	2-

IQR, Interquartile range; SD, Standard deviation; NCRD, non-chronic respiratory disease; CRD, chronic respiratory disease

In the 101 included studies, we identified 1,229,434 patients with COVID-19, and 32,301, 10,827, and 818 had asthma, COPD, and ACO, respectively, as the comorbidities. Among the studies, there were 34 reports from USA, 14 from China, 10 from Italy, 8 from Spain, 6 from the UK, 5 from Turkey, 4 from Mexico, 3 from Korea, 2 from the Netherlands, 2 from Iran, and 1 each from Israel, Nigeria, Russia, Norway, and Columbia. The study designs were 52 retrospective studies, 7 prospective studies, 1 population-based statistics, 2 matched cohort studies, 4 longitudinal cohort studies, 2 local institutional reviews, 1 randomized, controlled trial, 2 nation cohort studies, 1 descriptive study, 3 case–control studies, 2 cross-sectional studies, and 24 with an unknown design. The total number of male patients was 616,380 and that of female patients was 737,188. Among the studies, the severity of patients with COVID varied from asymptomatic to a critical condition.

### Frequency of asthma, COPD, and ACO in patients with COVID-19

**Fig 2:**
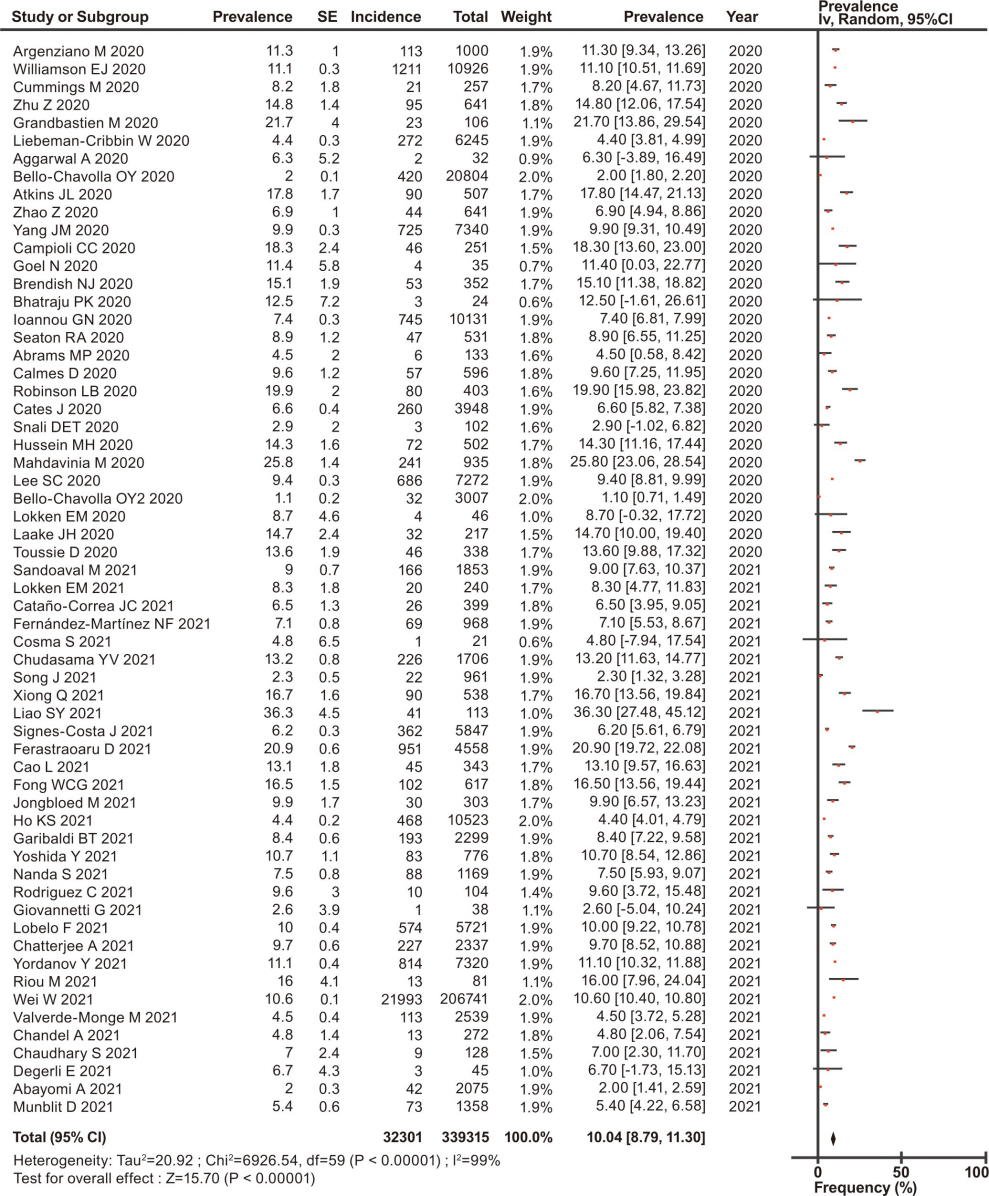
Forest plots of the prevalence of asthma in patients with COVID-19.

**Fig 3:**
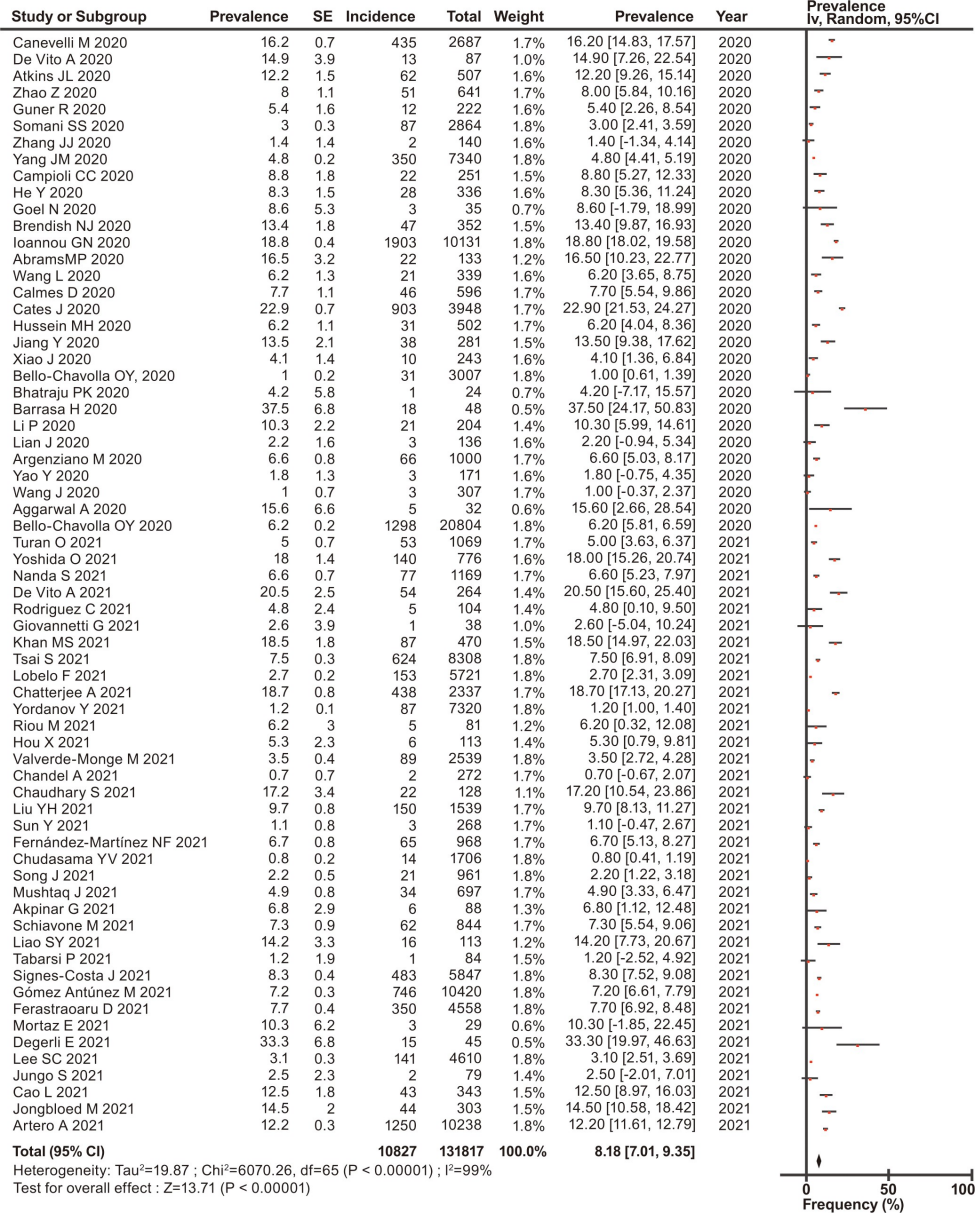
Forest plots of the prevalence of COPD in patients with COVID-19.

**Fig 4:**
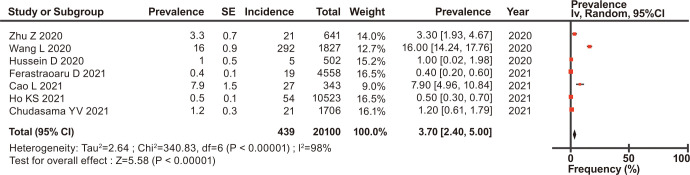
Forest plots of the prevalence of ACO in patients with COVID-19.

The overall prevalence of asthma, COPD, and ACO was estimated, and their forest plots are shown in Figs 2–4, respectively.

Among the eligible patients with COVID-19, the prevalence of asthma, COPD, and ACO was 10.04% (95% CI, 8.79–11.30) for asthma (Fig 2), 8.18% (95% CI, 7.01–9.35) for COPD (Fig 3), and 3.70% (95% CI, 2.40–5.00) for ACO (Fig 4). In the stratified analysis, the frequencies of asthma in different countries are shown in Table 2, and their forest plots are shown in S1 Fig.

**Table 2:** Estimated frequencies of asthma, COPD, and ACO in patients with COVID-19 according to countries.

	Asthma			COPD			ACO		
Country	No. of studies	No. of patients	Estimated frequency (95% CI)	No. of studies	No. of patients	Estimated frequency (95% CI)	No. of studies	No. of patients	Estimated frequency (95% CI)
USA	28	26692	11.14 (9.55–12.73)	19	4600	10.48 (7.56–13.40)	6	418	4.24 (2.74–5.73)
Mexico	2	452	1.57 (0.69–2.45)	2	1329	3.60 (-1.50–8.70)	-	-	-
UK	6	1729	13.45 (11.23–15.66)	3	123	8.69 (-0.83–18.22)	1	21	1.20 (0.61–1.79)
Italy	3	28	0.10 (0.09-.011)	6	599	11.09 (5.64–16.54)	-	-	-
Spain	3	544	5.83 (4.44–7.23)	6	2651	8.84 (5.77–11.91)	-	-	-
France	4	860	13.50 (9.08–17.92)	4	99	2.60 (0.33–4.88)	-	-	-
Netherland	2	257	9.72 (8.61–10.83)	2	482	17.00 (12.96–21.04)	-	-	-
Turkey	2	6	3.58 (0.02–7.13)	4	86	8.23 (3.47–12.98)	-	-	-
Iran	-	-	-	2	4	3.84 (-4.25–11.93)	-	-	-
India	2	6	8.57 (0.98–16.16)	2	8	11.34 (3.24–19.44)	-	-	-
China	2	112	9.42 (-4.69–23.53)	13	309	4.93 (2.89–6.96)	-	-	-
Korea	2	1411	9.65 (9.16–10.14)	2	491	3.96 (2.30–5.63)	-	-	-

With regard to the frequency of asthma, France showed a rate of 13.50% (95% CI, 9.08–17.92), which was the highest, followed by 13.45% in the UK (95% CI, 11.23–15.66). The frequency of COPD in patients with COVID-19 was the highest in the Netherlands at 17.00% (95% CI, 12.96–21.04), followed by India at 11.34% (95% CI, 3.24–19.44). The frequency of ACO on the USA and the UK was 4.24% (95% CI, 2.74–5.73) and 1.20% (95% CI, 0.61–1.79), respectively. The forest plots of these data are shown in supplementary figures (S1 Fig).

### Prevalence of death in patients with COVID-19 and asthma or COPD

**Fig 5:**
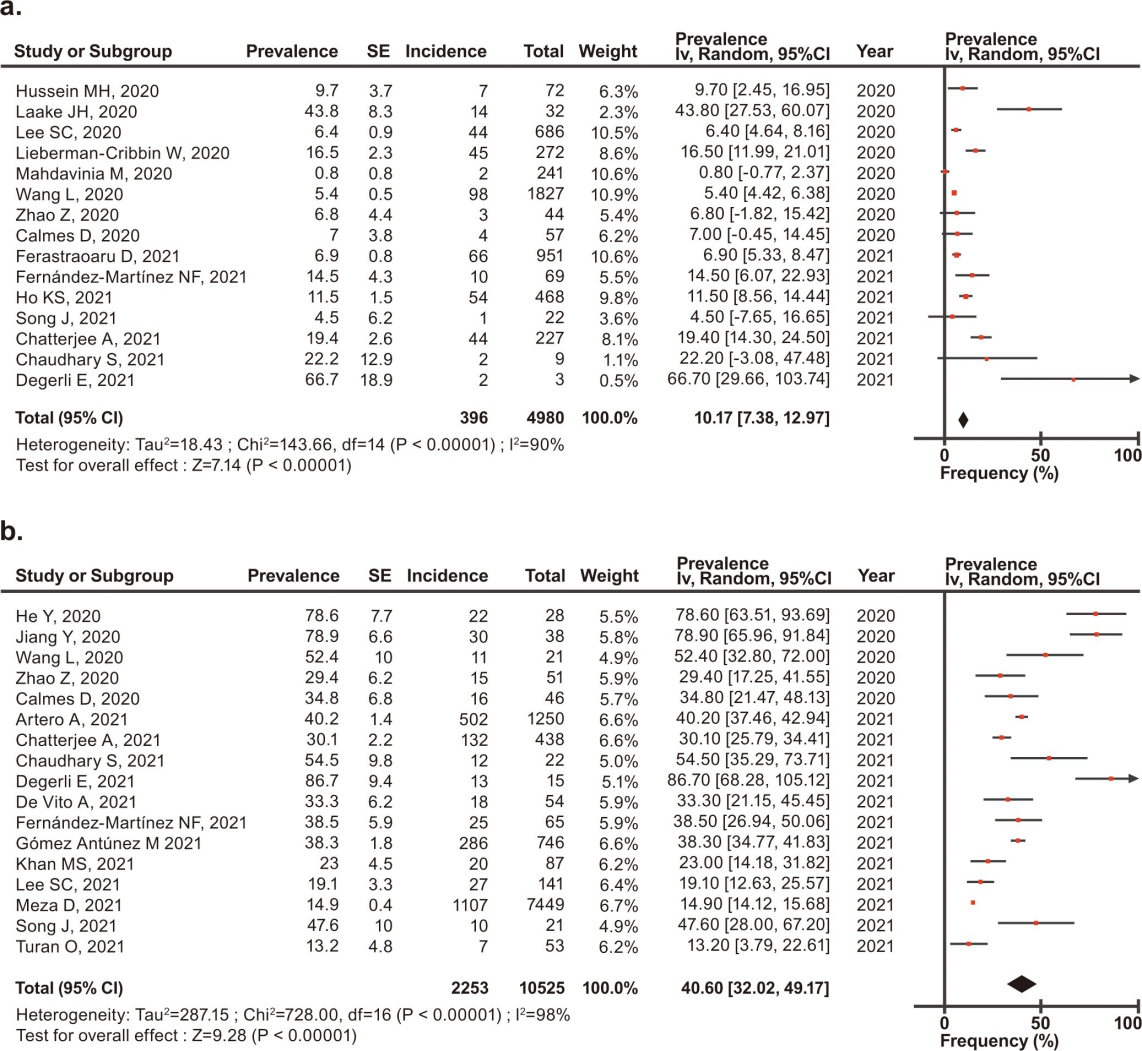
Forest plots of the prevalence of death a) in patients with asthma and COVID-19 and b) in patients with COPD and COVID-19.

Forest plots of the prevalence of death in patients with COVID-19 and asthma or COPD are shown in Fig 5A and 5B.

Among 4,980 patients with asthma and COVID-19, the prevalence of death was 10.17% (95% CI, 7.38–12.97) (Fig 5A). Among 10,525 patients with COPD and COVID-19, the prevalence of death was 40.60% (95% CI, 32.02–49.17) (Fig 5B).

### Risk of mortality due to COVID-19 in patients with asthma or COPD

**Fig 6:**
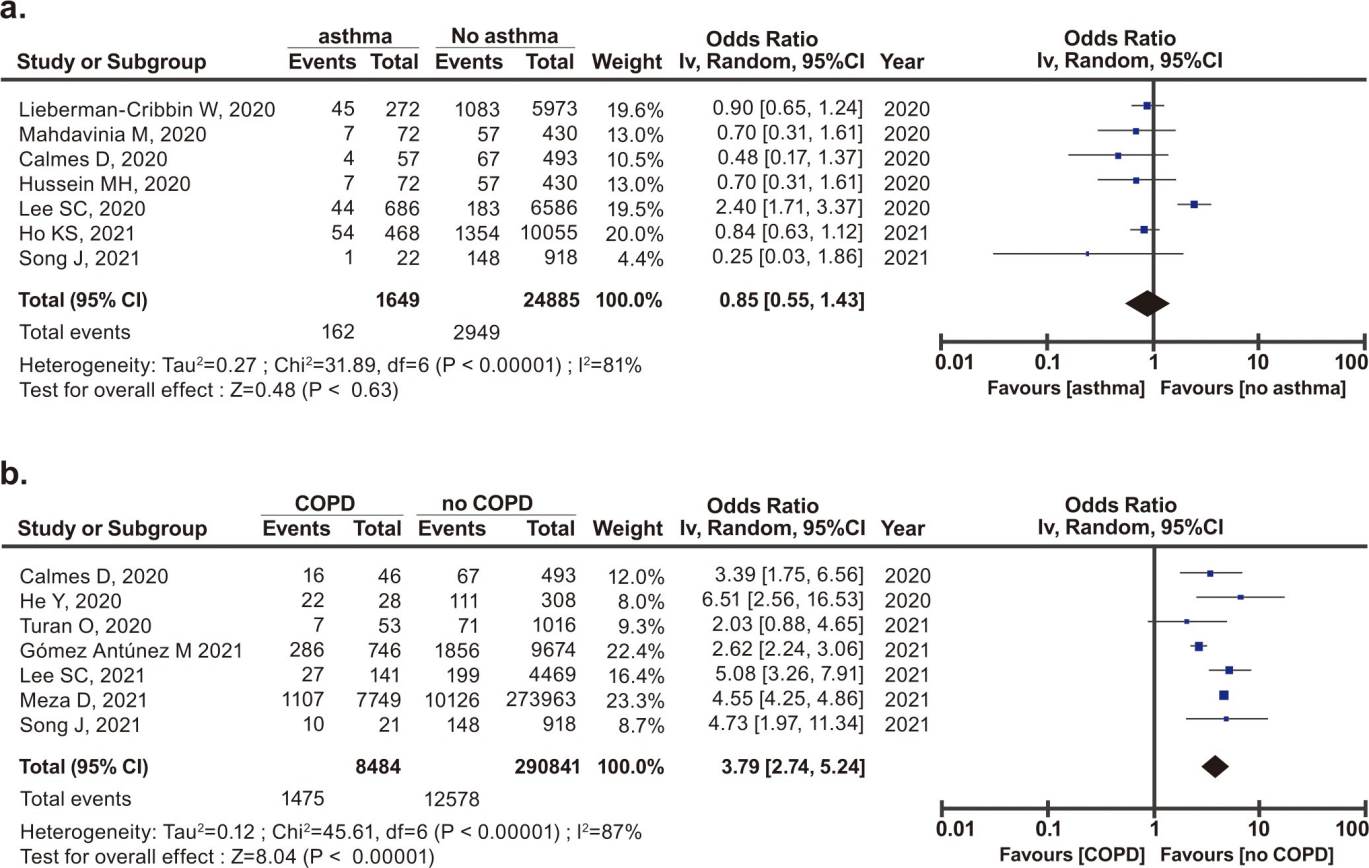
Forest plots of the risk of mortality in patients with COVID-19 and a) asthma or b) COPD.

The risk to mortality due to COVID-19 in patients with asthma or COPD was estimated and it is shown in forest plots in Fig 6.

The risk of mortality in pre-existing asthma in COVID-19 patients was not significant (OR, 0.89; 95% CI, 0.55–1.43; *p* = 0.630) (Fig 6A). However, the risk of mortality in pre-existing COPD in COVID-19 patients was significant (OR, 3.79; 95% CI, 2.74–5.24; *p*<0.001) (Fig 6B).

## Discussion

The present systematic review and meta-analysis on 101 studies showed that pre-existing asthma and COPD affected the incidence of COVID-19, and asthma had a greater effect than COPD. However, pre-existing asthma did not have a significant effect on mortality in patients with COVID-19, while patients with COPD had a 3.8-fold increased risk of mortality among COVID-19 cases. Among patients with COVID-19, the highest prevalence of asthma was observed in France followed by the UK, while the highest prevalence of COPD was observed in the Netherlands followed by India. The various prevalence of these disease in each county indicates the importance of daily clinical control of asthma, COPD, and ACO for preventing and reducing the severity of COVID-19.

The COVID-19 pandemic has disproportionately affected people with chronic diseases, such as asthma and COPD, which are the most common respiratory diseases. Generally, viral infection to the respiratory tract is thought to be one of the triggers for the exacerbation of pre-existing diseases [110]. Respiratory viral infection that is initiated in the upper respiratory tract and innate immunity are critical for the initial control of infection at this site [122]. If the innate immune response is inadequate, the infection can spread to the lower respiratory tract, causing pneumonia [123]. Before the COVID-19 pandemic, the reported global prevalence of adult asthma and COPD was 3.5% [9] and 12.16% [10], respectively. However, in patients with COVID-19 in the present study, which assessed studies published after COVID-19 emerged, the prevalence of pre-existing asthma was 10.04% and that of COPD was 8.18%. The prevalence of asthma after COVID-19 emerged was higher than that before the pandemic. These results indicated that asthma affected the incidence of COVID-19. The increased susceptibility of viral infection in the bronchial airway might be caused by pathophysiological impairment in both of these diseases. Especially in asthma, the main involved sites of the bronchial airway are the upper and lower bronchi [124]. In case of COVID-19, more than 80% of patients have mild illness [123], and the locations where mild COVID-19 is involved are similar to those in patients with asthma. Consequently, the number of patients with asthma may have increased as the number and proportion of mild COVID-19 cases increased. This possibility may also explain why the prevalence of pre-existing asthma was higher than that of COPD. In fact, the Omicron variant was associated with a large number of mild COVID-19 cases [125]. Additionally, a previous meta-analysis, which used only data before the Omicron variant emerged, reported that the prevalence of asthma was similar to that before the COVID-19 pandemic [126]. This result is different from that in the present study, which assessed COVID-19 cases that included infected patients with the Omicron variant. However, a study including hospitalized COVID-19 cases with a history of asthma indicated that none of these patients presented with asthma exacerbation [127]. Owing to the nature of the meta-analysis, we could not evaluate asthma exacerbation after admission among the patients in this study.

The present study showed that pre-existing COPD in patients had a 3.8-fold higher risk of mortality than in those who did not have COPD. The risk of mortality for pre-existing COPD was stronger than that for pre-existing asthma. Unlike asthma, of which the main involved sites are the upper and lower bronchi, the main impaired lesion of COPD extends from the peripheral small airway to alveolar tissues with architectural damage, which can cause the severe illness. These locations of lesions are compatible with those in COVID-19 when the disease severity is moderate to severe. Indeed, patients with COPD have a high risk of mortality in other respiratory infectious diseases, such as influenza [128] and community-acquired pneumonia [129]. A previous study showed that the long-term use of inhaled corticosteroids for controlling asthma is likely to have a beneficial modulatory effect on COVID-19 [130]. This finding suggests that this efficacy is achieved by reducing epithelial damage and improving the T-cell response. Several studies reported a large number of patients who were receiving either inhaled steroids or systemic steroids at the time of COVID-19 diagnosis [55, 127, 131, 132]. However, the effect of inhaled corticosteroids at the early stage of COVID-19 is controversial [133]. The benefit of systemic corticosteroids for patients with asthma may outweigh the risk of severe outcomes in patients with COVID-19 [134]. Systemic corticosteroids are effective for treating bronchial wall inflammation and bronchial spasm. As the result, uncontrolled asthma is associated with increased intensive care unit admission and intensive respiratory support [135], whereas well-controlled asthma does not have an increased risk of COVID-19-related death [136]. The present study showed that the prevalence of pre-existing asthma in COVID-19 cases varied according to the countries. This finding may be partly due to the fact that each country has different treatment policies and guidelines, as well as available medical resources. In addition, owing to the nature of the meta-analysis in which we did not use individual patient data, we were unable to examine the impact on COVID-19 diagnosis according to age, sex, and stage at which therapy was started. These differences may also influence the severity of COVID-19 in different countries. These factors may be also related to the heterogeneity in the results of the meta-analysis in the present study. A large-sample study showed that the contribution of inhaled corticosteroids for patients with COPD to COVID-19-related death was lower than that for patients with asthma [137]. Additionally, the association with mortality was confounded by the presence of other risk factors for severe COVID-19, such as an older age, cardiovascular disease, hypertension, and diabetes mellitus [123], which are common in people with COPD.

ACO has clinical characteristics derived from asthma and COPD. The risk of mortality from ACO in patients with COVID-19 might be significant and as high as that for COPD. However, in the present study, the prevalence of pre-existing ACO was lower than that of asthma and COPD. Our results regarding ACO cannot be properly assessed because of the number of eligible studies, and the countries that reported pre-existing ACO were only from the USA, UK, and China among the eligible studies. These issues might be due to the short history of the concept of ACO and a lack of global recognition. However, even with the small number of eligible studies, the prevalence of ACO was highest in studies from the USA. Additionally, a study in the USA before the COVID-19 pandemic reported that the prevalence of ACO was 1.05% (0.74%–1.37%) [138], while that in the present study was 4.24%. One of the reasons for this discrepancy between studies may be related to the high smoking rate (14%) in the USA [139–141]. This discrepancy suggests the necessity of considering other cofounding factors for assessing the risk of ACO, such as the rate of smokers and obesity.

Our study has some limitations, including mainly those inherent to the nature of systematic reviews and meta-analyses using observational studies and case series. The eligible studies were limited to articles written in English. The treatment guidelines and available medical resources for COVID-19, and the examined comorbidities may be different according to the different countries, and these could have affected the risk of infection and mortality of COVID-19. The eligible studies were selected from published papers during 1 year and 9 months from the beginning of the COVID-19 pandemic. We were not able to evaluate the change in the risk of COVID-19 caused by the change in SARS-CoV-2 variants and the vaccination availability during this observational period.

Despite these limitations, the present systematic review and meta-analysis of 101 studies suggests the importance of daily clinical management for patients with asthma, COPD, or ACO. Additionally, this study suggests that attention should be paid to the prevention of COVID-19 infection and disease progression, as well as to patients with other high-risk diseases of COVID-19.

## Conclusion

The present systematic review and meta-analysis using 101 studies shows that pre-existing asthma and COPD are associated with the incidence of COVID-19. Asthma has a stronger influence on the incidence of COVID-19 than COPD. The presence of COPD as a comorbidity in patients with COVID-19 has a 3.8 times higher risk of mortality, while asthma has no significant effect on COVID-19 related death. These differences appear to be affected by the difference in locations of disease pathophysiology, and by the daily diagnosis and treatment policy of each country.

## Supporting information

S1 Checklist (DOCX)

S1 Table Classification standard of the evidence level. (DOCX)

S1 Fig Forrest plots for prevalence of asthma among patients with COVID-19. (PDF)

S2 Fig Forrest plots for prevalence of COPD among patients with COVID-19. (PDF)

S3 Fig Forrest plots for prevalence of ACO among patients with COVID-19. (PDF)
