# Supplementary material for: Effect of asthma, COPD, and ACO on COVID-19: A systematic review and meta-analysis
Source: PLoS One. 2022 Nov 1;17(11):e0276774. doi: 10.1371/journal.pone.0276774 (PMC9624422; doi:10.1371/journal.pone.0276774)
Supplement: S1 Table — (DOCX) [file pone.0276774.s002.docx]

**S1 Table. Classification standard of the evidence level.**

| Level of Evidence | Study |
| --- | --- |
| 1++ | High quality of meta-analysis, systematic reviews of RCTs, or RCTs with a very low risk of bias |
| 1+ | Well controlled meta-analysis, systematic reviews, or low risk of bias |
| 1- | Meta-analysis, systematic reviews or RCTs, or RCTs with a high risk of bias |
| 2++ | High quality of systematic reviews of case-control or cohort studies  Or High quality of case-control studies with a low risk of confounding, bias or chance and a high probability that the relationship is causal |
| 2+ | Well controlled case-control or cohort studies with a low risk of confounding, bias or chance and a moderate probability that the relationship is causal |
| 2- | Case-control or cohort studies with a high risk of confounding, bias or chance and a moderate probability that the relationship is not causal |
| 3 | Non-analytic studies, eg case reports, case series |
| 4 | Expert opinion |

RCT, randomized controlled trial
