# Supplementary material for: Effect of asthma, COPD, and ACO on COVID-19: A systematic review and meta-analysis
Source: PLoS One. 2022 Nov 1;17(11):e0276774. doi: 10.1371/journal.pone.0276774 (PMC9624422; doi:10.1371/journal.pone.0276774)

S1 Fig. Forrest plots for prevalence of asthma among patients with COVID-19.

a. USA

Study or Subgroup	Prevalence	SE	Incidence	Total	Weight	Prevalence	Year
Zhao Z 2020	6.9	1	44	641	4.0%	6.90 [4.94, 8.86]	2020
Campioli CC 2020	18.3	2.4	46	251	3.1%	18.30 [13.60, 23.00]	2020
Bhatraju PK 2020	12.5	7.2	3	24	1.0%	12.50 [-1.61, 26.61]	2020
Ioannou GN 2020	7.4	0.3	745	10131	4.2%	7.40 [6.81, 7.99]	2020
Abrams MP 2020	4.5	2	6	133	3.4%	4.50 [0.58, 8.42]	2020
Robinson LB 2020	19.9	2	80	403	3.4%	19.90 [15.98, 23.82]	2020
Cates J 2020	6.6	0.4	260	3948	4.2%	6.60 [5.82, 7.38]	2020
Hussein MH 2020	14.3	1.6	72	502	3.6%	14.30 [11.16, 17.44]	2020
Mahdavinia M 2020	25.8	1.4	241	935	3.8%	25.80 [23.06, 28.54]	2020
Lokken EM 2020	8.7	4.6	4	46	1.8%	8.70 [-0.32, 17.72]	2020
Toussie D 2020	13.6	1.9	46	338	3.4%	13.60 [9.88, 17.32]	2020
Argenziano M 2020	11.3	1	113	1000	4.0%	11.30 [9.34, 13.26]	2020
Cummings MJ 2020	8.2	1.8	21	257	3.5%	8.20 [4.67, 11.73]	2020
Zhu Z 2020	14.8	1.4	95	641	3.8%	14.80 [12.06, 17.54]	2020
Lieberman-Cribbin W 2020	4.4	0.3	272	6245	4.2%	4.40 [3.81, 4.99]	2020
Liao SY 2021	36.3	4.5	41	113	1.8%	36.30 [27.48, 45.12]	2021
Ferastraoaru D 2021	20.9	0.6	951	4558	4.1%	20.90 [19.72, 22.08]	2021
Cao L 2021	13.1	1.8	45	343	3.5%	13.10 [9.57, 16.63]	2021
Ho KS 2021	4.4	0.2	468	10523	4.2%	4.40 [4.01, 4.79]	2021
Garibaldi BT 2021	8.4	0.6	193	2299	4.1%	8.40 [7.22, 9.58]	2021
Yoshida Y 2021	10.7	1.1	83	776	3.9%	10.70 [8.54, 12.86]	2021
Nanda S 2021	7.5	0.8	88	1169	4.1%	7.50 [5.93, 9.07]	2021
Lobelo F 2021	10	0.4	574	5721	4.2%	10.00 [9.22, 10.78]	2021
Wei W 2021	10.6	0.1	21993	206741	4.2%	10.60 [10.40, 10.80]	2021
Chandel A 2021	4.8	1.4	13	272	3.8%	4.80 [2.06, 7.54]	2021
Chaudhary S 2021	7	2.4	9	128	3.1%	7.00 [2.30, 11.70]	2021
Sandoaval M 2021	9	0.7	166	1853	4.1%	9.00 [7.63, 10.37]	2021
Lokken EM 2021	8.3	1.8	20	240	3.5%	8.30 [4.77, 11.83]	2021
**Total (95% CI)**			**26692**	**260231**	**100.0%**	**11.14 [9.55, 12.73]**	

Heterogeneity: Tau² = 15.46; Chi² = 1779.40, df = 27 (P < 0.00001); I² = 98%
Test for overall effect: Z = 13.76 (P < 0.00001)

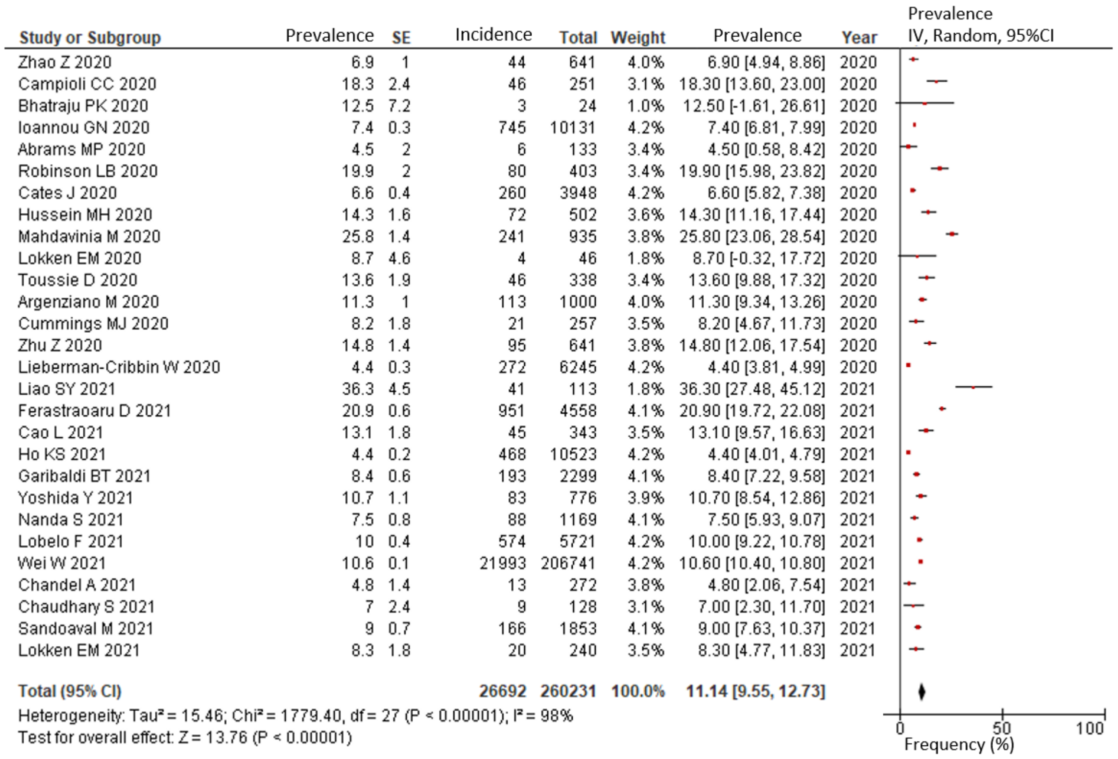

b. Mexico

Study or Subgroup	Prevalence	SE	Incidence	Total	Weight	Prevalence IV, Random, 95% CI	Year
Bello-Chavolla OY 2020	2	0.1	420	20804	51.9%	2.00 [1.80, 2.20]	2020
Bello-Chavolla OY2 2020	1.1	0.2	32	3007	48.1%	1.10 [0.71, 1.49]	2020
**Total (95% CI)**			**452**	**23811**	**100.0%**	**1.57 [0.69, 2.45]**	

Heterogeneity: Tau² = 0.38; Chi² = 16.20, df = 1 (P < 0.0001); I² = 94%
Test for overall effect: Z = 3.48 (P = 0.0005)

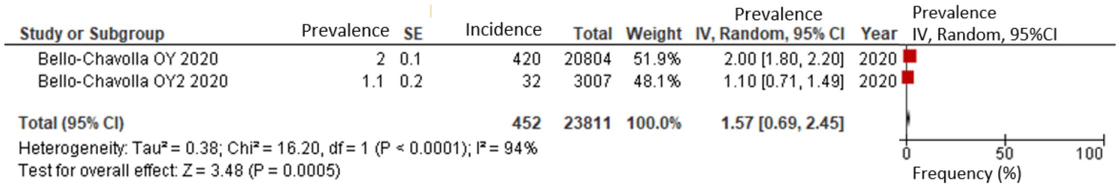

c. UK

Study or Subgroup	Prevalence	SE	Incidence	Total	Weight	Prevalence IV, Random, 95% CI	Year
Atkins JL 2020	17.8	1.7	90	507	14.3%	17.80 [14.47, 21.13]	2020
Brendish NJ 2020	15.1	1.9	53	352	13.2%	15.10 [11.38, 18.82]	2020
Seaton RA 2020	8.9	1.2	47	531	17.1%	8.90 [6.55, 11.25]	2020
Williamson EJ 2020	11.1	0.3	1211	10926	20.8%	11.10 [10.51, 11.69]	2020
Fong WCG 2021	16.5	1.5	102	617	15.4%	16.50 [13.56, 19.44]	2021
Chudasama YV 2021	13.2	0.8	226	1706	19.1%	13.20 [11.63, 14.77]	2021
**Total (95% CI)**			**1729**	**14639**	**100.0%**	**13.45 [11.23, 15.66]**	

Heterogeneity: Tau² = 6.05; Chi² = 39.20, df = 5 (P < 0.00001); I² = 87%
Test for overall effect: Z = 11.89 (P < 0.00001)

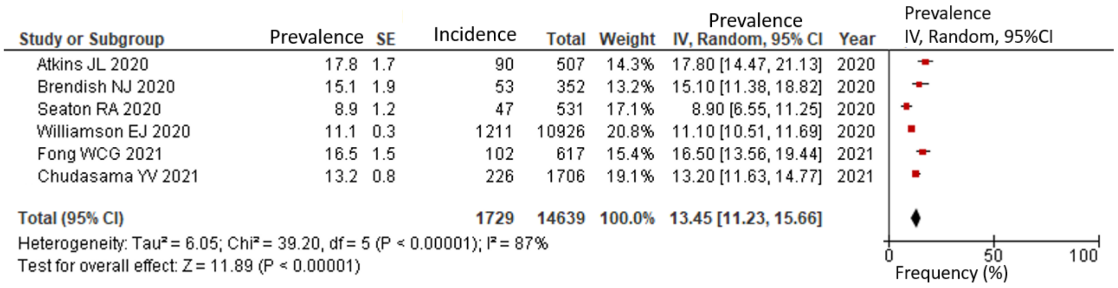

d. Italy

Study or Subgroup	Prevalence	SE	Incidence	Total	Weight	Prevalence IV, Random, 95% CI	Year
Caminati M 2020	0.1	0.005	26	17540	100.0%	0.10 [0.09, 0.11]	2020
Giovannetti G 2021	2.6	3.9	1	38	0.0%	2.60 [-5.04, 10.24]	2021
Cosma S 2021	4.8	6.5	1	21	0.0%	4.80 [-7.94, 17.54]	2021
**Total (95% CI)**			**28**	**17599**	**100.0%**	**0.10 [0.09, 0.11]**	

Heterogeneity: Tau² = 0.00; Chi² = 0.93, df = 2 (P = 0.63); I² = 0%
Test for overall effect: Z = 20.00 (P < 0.00001)

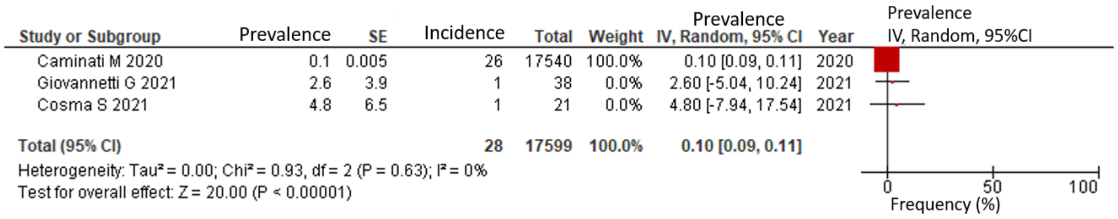

e. Spain

Study or Subgroup	Prevalence	SE	Incidence	Total	Weight	Prevalence IV, Random, 95% CI	Year
Signes-Costa J 2021	6.2	0.3	362	5847	37.6%	6.20 [5.61, 6.79]	2021
Valverde-Monge M 2021	4.5	0.4	113	2539	35.7%	4.50 [3.72, 5.28]	2021
Fernández-Martínez NF 2021	7.1	0.8	69	968	26.7%	7.10 [5.53, 8.67]	2021
**Total (95% CI)**			**544**	**9354**	**100.0%**	**5.83 [4.44, 7.23]**	

Heterogeneity: Tau² = 1.26; Chi² = 14.84, df = 2 (P = 0.0006); I² = 87%
Test for overall effect: Z = 8.20 (P < 0.00001)

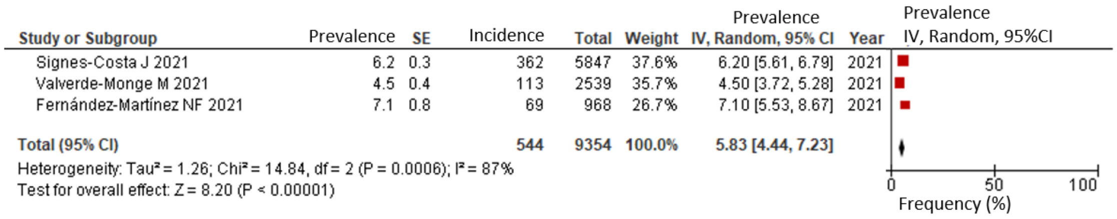

f. France

Study or Subgroup	Prevalence	SE	Incidence	Total	Weight	Prevalence IV, Random, 95% CI	Year
Grandbastien M 2020	21.7	4	23	106	18.0%	21.70 [13.86, 29.54]	2020
Rodriguez C 2021	9.6	3	10	104	23.9%	9.60 [3.72, 15.48]	2021
Yordanov Y 2021	11.1	0.4	814	7320	40.7%	11.10 [10.32, 11.88]	2021
Riou M 2021	16	4.1	13	81	17.5%	16.00 [7.96, 24.04]	2021
**Total (95% CI)**			**860**	**7611**	**100.0%**	**13.50 [9.08, 17.92]**	

Heterogeneity: Tau² = 12.33; Chi² = 8.61, df = 3 (P = 0.04); I² = 65%
Test for overall effect: Z = 5.99 (P < 0.00001)

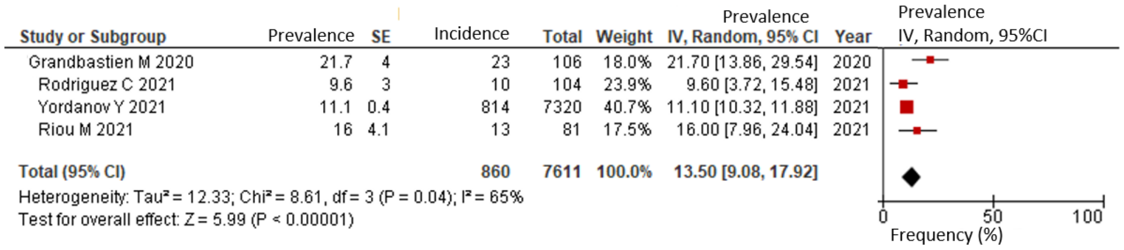

g. Netherland

Study or Subgroup	Prevalence	SE	Incidence	Total	Weight	Prevalence IV, Random, 95% CI	Year
Jongbloed M 2021	9.9	1.7	30	303	11.1%	9.90 [6.57, 13.23]	2021
Chatterjee A 2021	9.7	0.6	227	2337	88.9%	9.70 [8.52, 10.88]	2021
**Total (95% CI)**			**257**	**2640**	**100.0%**	**9.72 [8.61, 10.83]**	

Heterogeneity: Tau² = 0.00; Chi² = 0.01, df = 1 (P = 0.91); I² = 0%
Test for overall effect: Z = 17.18 (P < 0.00001)

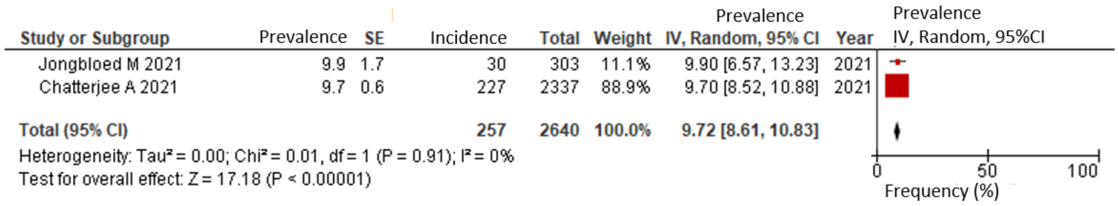

h. China

Study or Subgroup	Prevalence	SE	Incidence	Total	Weight	Prevalence IV, Random, 95% CI	Year
Song J 2021	2.3	0.5	22	961	50.6%	2.30 [1.32, 3.28]	2021
Xiong Q 2021	16.7	1.6	90	538	49.4%	16.70 [13.56, 19.84]	2021
**Total (95% CI)**			**112**	**1499**	**100.0%**	**9.42 [-4.69, 23.53]**	

Heterogeneity: Tau² = 102.27; Chi² = 73.79, df = 1 (P < 0.00001); I² = 99%
Test for overall effect: Z = 1.31 (P = 0.19)

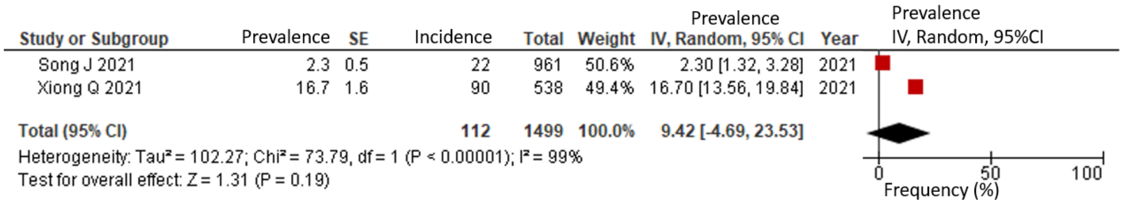

i. India

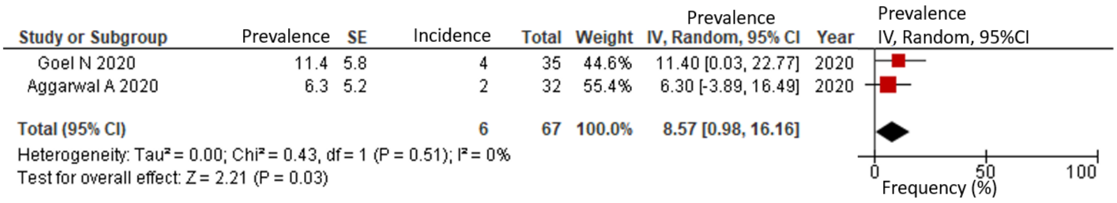

j. Turkey

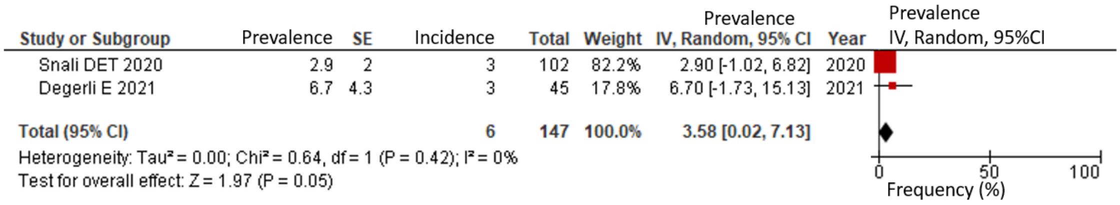

k. Korea

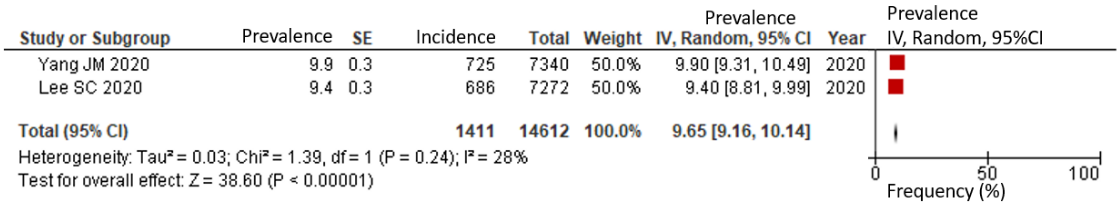

Supplement: S1 Fig — (PDF) [file pone.0276774.s003.pdf]
