# Supplementary material for: Effect of asthma, COPD, and ACO on COVID-19: A systematic review and meta-analysis
Source: PLoS One. 2022 Nov 1;17(11):e0276774. doi: 10.1371/journal.pone.0276774 (PMC9624422; doi:10.1371/journal.pone.0276774)

S2 Fig. Forrest plots for prevalence of COPD among patients with COVID-19.

a. USA

Study or Subgroup	Prevalence	SE	Incidence	Total	Weigh	Prevalence	Year
Bhatraju PK 2020	4.2	5.8	1	24	3.1%	4.20 [-7.17, 15.57]	2020
Argenziano M 2020	6.6	0.8	66	1000	5.6%	6.60 [5.03, 8.17]	2020
Zhao Z 2020	8	1.1	51	641	5.6%	8.00 [5.84, 10.16]	2020
Somani SS 2020	3	0.3	87	2864	5.7%	3.00 [2.41, 3.59]	2020
Campioli CC 2020	8.8	1.8	22	251	5.3%	8.80 [5.27, 12.33]	2020
Ioannou GN 2020	18.8	0.4	1903	10131	5.7%	18.80 [18.02, 19.58]	2020
Abrams MP 2020	16.5	3.2	22	133	4.5%	16.50 [10.23, 22.77]	2020
Cates J 2020	22.9	0.7	903	3948	5.7%	22.90 [21.53, 24.27]	2020
Hussein MH 2020	6.2	1.1	31	502	5.6%	6.20 [4.04, 8.36]	2020
Chandel A 2021	0.7	0.7	2	272	5.7%	0.70 [-0.67, 2.07]	2021
Chaudhary S 2021	17.2	3.4	22	128	4.4%	17.20 [10.54, 23.86]	2021
Liao SY 2021	14.2	3.3	16	113	4.5%	14.20 [7.73, 20.67]	2021
Ferastraoaru D 2021	7.7	0.4	350	4558	5.7%	7.70 [6.92, 8.48]	2021
Cao L 2021	12.5	1.8	43	343	5.3%	12.50 [8.97, 16.03]	2021
Yoshida Y 2021	18	1.4	140	776	5.4%	18.00 [15.26, 20.74]	2021
Nanda S 2021	6.6	0.7	77	1169	5.7%	6.60 [5.23, 7.97]	2021
Khan MS 2021	18.5	1.8	87	470	5.3%	18.50 [14.97, 22.03]	2021
Tsai S 2021	7.5	0.3	624	8308	5.7%	7.50 [6.91, 8.09]	2021
Lobelo F 2021	2.7	0.2	153	5721	5.7%	2.70 [2.31, 3.09]	2021
**Total (95% CI)**			**4600**	**41352**	**100.0%**	**10.48 [7.56, 13.40]**	

Heterogeneity: Tau² = 38.75; Chi² = 2232.23, df = 18 (P < 0.00001); I² = 99%
Test for overall effect: Z = 7.04 (P < 0.00001)

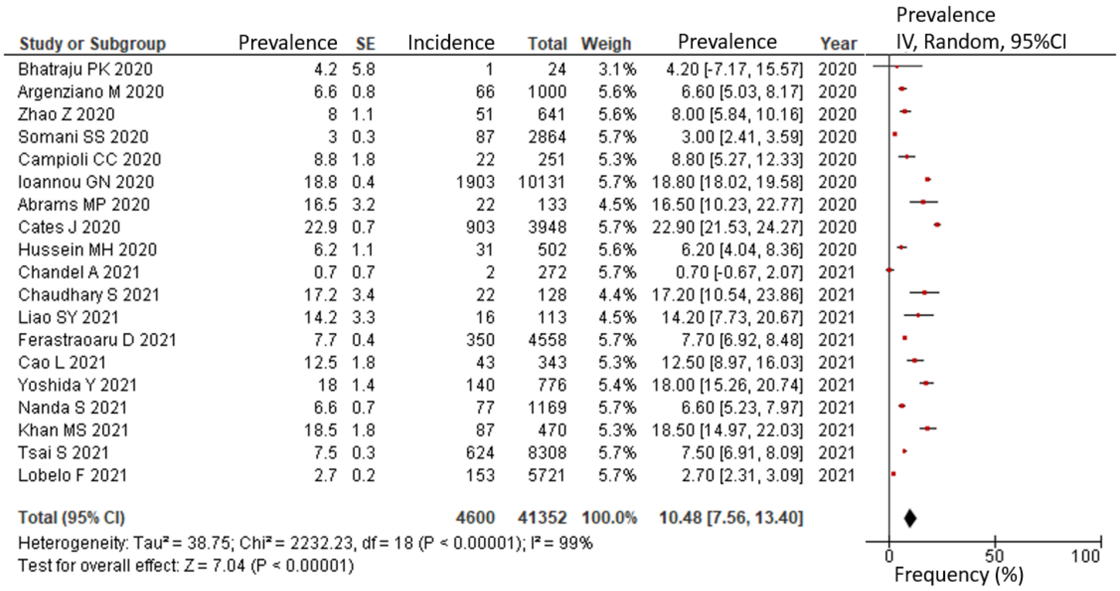

b. Mexico

Study or Subgroup	Prevalence	SE	Incidence	Total	Weight	Prevalence IV, Random, 95% CI	Year
Bello-Chavolla OY 2020	6.2	0.2	1298	20804	50.0%	6.20 [5.81, 6.59]	2020
Bello-Chavolla OY, 2020	1	0.2	31	3007	50.0%	1.00 [0.61, 1.39]	2020
**Total (95% CI)**			**1329**	**23811**	**100.0%**	**3.60 [-1.50, 8.70]**	

Heterogeneity: Tau² = 13.48; Chi² = 338.00, df = 1 (P < 0.00001); I² = 100%
Test for overall effect: Z = 1.38 (P = 0.17)

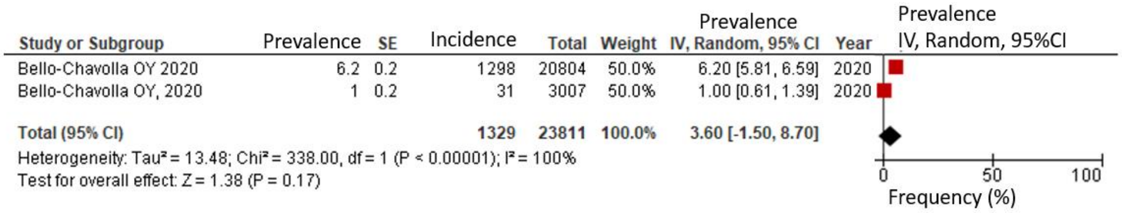

c. UK

Study or Subgroup	Prevalence	SE	Incidence	Total	Weight	Prevalence IV, Random, 95% CI	Year
Atkins JL 2020	12.2	1.5	62	507	33.1%	12.20 [9.26, 15.14]	2020
Brendish NJ 2020	13.4	1.8	47	352	32.7%	13.40 [9.87, 16.93]	2020
Chudasama YV 2021	0.8	0.2	14	1706	34.2%	0.80 [0.41, 1.19]	2021
**Total (95% CI)**			**123**	**2565**	**100.0%**	**8.69 [-0.83, 18.22]**	

Heterogeneity: Tau² = 69.02; Chi² = 103.65, df = 2 (P < 0.00001); I² = 98%
Test for overall effect: Z = 1.79 (P = 0.07)

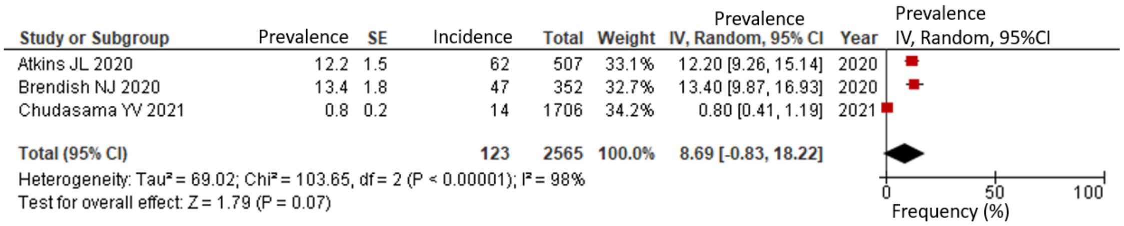

d. Spain

Study or Subgroup	Prevalence	SE	Incidence	Total	Weight	Prevalence IV, Random, 95% CI	Year
Barrasa H 2020	37.5	6.8	18	48	4.2%	37.50 [24.17, 50.83]	2020
Fernández-Martínez NF 2021	6.7	0.8	65	968	18.6%	6.70 [5.13, 8.27]	2021
Signes-Costa J 2021	8.3	0.4	483	5847	19.3%	8.30 [7.52, 9.08]	2021
Gómez Antúnez M 2021	7.2	0.3	746	10420	19.4%	7.20 [6.61, 7.79]	2021
Artero A 2021	12.2	0.3	1250	10238	19.4%	12.20 [11.61, 12.79]	2021
Valverde-Monge M 2021	3.5	0.4	89	2539	19.3%	3.50 [2.72, 4.28]	2021
**Total (95% CI)**			**2651**	**30060**	**100.0%**	**8.84 [5.77, 11.91]**	

Heterogeneity: Tau² = 12.60; Chi² = 348.87, df = 5 (P < 0.00001); I² = 99%
Test for overall effect: Z = 5.64 (P < 0.00001)

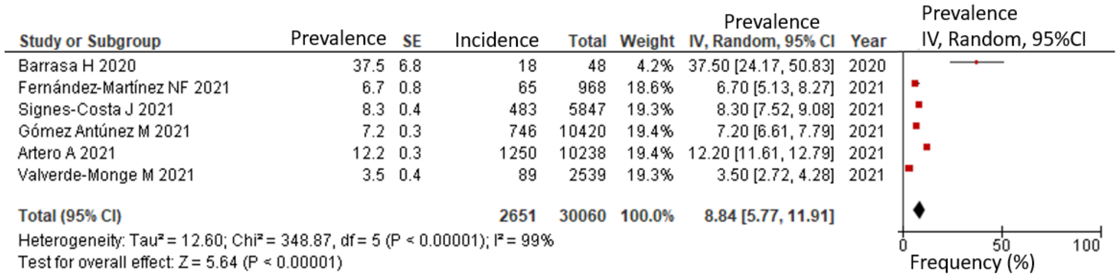

e. Italy

Study or Subgroup	Prevalence	SE	Incidence	Total	Weight	Prevalence IV, Random, 95% CI	Year
Canevelli M 2020	16.2	0.7	435	2687	18.7%	16.20 [14.83, 17.57]	2020
De Vito A 2020	14.9	3.9	13	87	13.8%	14.90 [7.26, 22.54]	2020
De Vito A 2021	20.5	2.5	54	264	16.4%	20.50 [15.60, 25.40]	2021
Giovannetti G 2021	2.6	3.9	1	38	13.8%	2.60 [-5.04, 10.24]	2021
Mushtaq J 2021	4.9	0.8	34	697	18.7%	4.90 [3.33, 6.47]	2021
Schiavone M 2021	7.3	0.9	62	844	18.6%	7.30 [5.54, 9.06]	2021
**Total (95% CI)**			**599**	**4617**	**100.0%**	**11.09 [5.64, 16.54]**	

Heterogeneity: Tau² = 40.79; Chi² = 149.31, df = 5 (P < 0.00001); I² = 97%
Test for overall effect: Z = 3.99 (P < 0.0001)

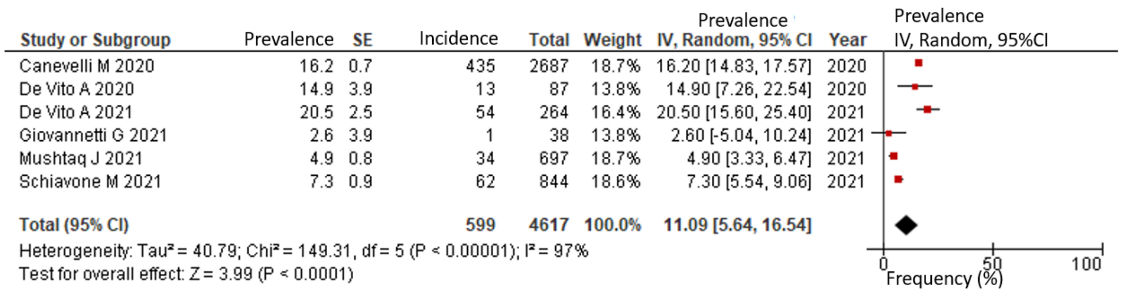

f. France

Study or Subgroup	Prevalence	SE	Incidence	Total	Weight	Prevalence IV, Random, 95% CI	Year
Yordanov Y 2021	1.2	0.1	87	7320	54.5%	1.20 [1.00, 1.40]	2021
Riou M 2021	6.2	3	5	81	11.8%	6.20 [0.32, 12.08]	2021
Jungo S 2021	2.5	2.3	2	79	17.4%	2.50 [-2.01, 7.01]	2021
Rodriguez C 2021	4.8	2.4	5	104	16.4%	4.80 [0.10, 9.50]	2021
**Total (95% CI)**			**99**	**7584**	**100.0%**	**2.60 [0.33, 4.88]**	

Heterogeneity: Tau² = 2.46; Chi² = 5.33, df = 3 (P = 0.15); I² = 44%
Test for overall effect: Z = 2.24 (P = 0.02)

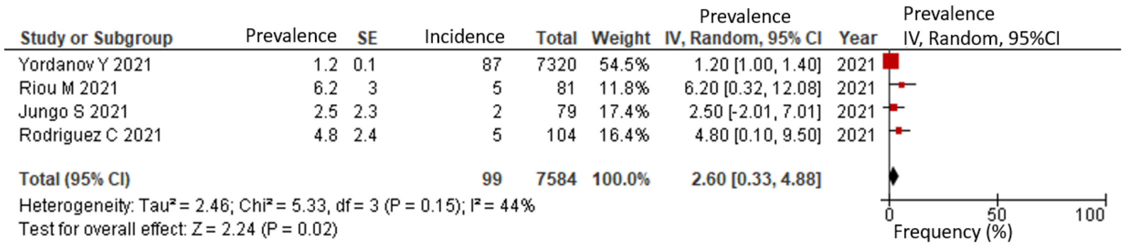

g. Netherland

Study or Subgroup	Prevalence	SE	Incidence	Total	Weight	Prevalence IV, Random, 95% CI	Year
Jongbloed M 2021	14.5	2	44	303	40.5%	14.50 [10.58, 18.42]	2021
Chatterjee A 2021	18.7	0.8	438	2337	59.5%	18.70 [17.13, 20.27]	2021
**Total (95% CI)**			**482**	**2640**	**100.0%**	**17.00 [12.96, 21.04]**	

Heterogeneity: Tau² = 6.50; Chi² = 3.80, df = 1 (P = 0.05); I² = 74%
Test for overall effect: Z = 8.25 (P < 0.00001)

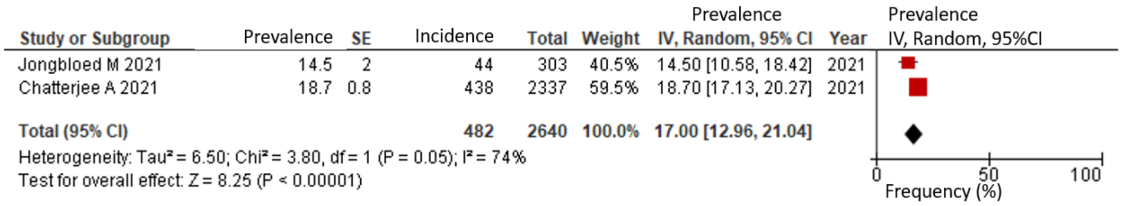

h. China

Study or Subgroup	Prevalence	SE	Incidence	Total	Weight	Prevalence IV, Random, 95% CI	Year
Zhang JJ 2020	1.4	1.4	2	140	7.7%	1.40 [-1.34, 4.14]	2020
He Y 2020	8.3	1.5	28	336	7.6%	8.30 [5.36, 11.24]	2020
Wang L 2020	6.2	1.3	21	339	7.9%	6.20 [3.65, 8.75]	2020
Jiang Y 2020	13.5	2.1	38	281	6.6%	13.50 [9.38, 17.62]	2020
Xiao J 2020	4.1	1.4	10	243	7.7%	4.10 [1.36, 6.84]	2020
Li P 2020	10.3	2.2	21	204	6.4%	10.30 [5.99, 14.61]	2020
Lian J 2020	2.2	1.6	3	136	7.4%	2.20 [-0.94, 5.34]	2020
Yao Y 2020	1.8	1.3	3	171	7.9%	1.80 [-0.75, 4.35]	2020
Wang J 2020	1	0.7	3	307	8.6%	1.00 [-0.37, 2.37]	2020
Sun Y 2021	1.1	0.8	3	268	8.5%	1.10 [-0.47, 2.67]	2021
Song J 2021	2.2	0.5	21	961	8.8%	2.20 [1.22, 3.18]	2021
Hou X 2021	5.3	2.3	6	113	6.2%	5.30 [0.79, 9.81]	2021
Liu YH 2021	9.7	0.8	150	1539	8.5%	9.70 [8.13, 11.27]	2021
**Total (95% CI)**			**309**	**5038**	**100.0%**	**4.93 [2.89, 6.96]**	

Heterogeneity: Tau² = 11.98; Chi² = 140.68, df = 12 (P < 0.00001); I² = 91%
Test for overall effect: Z = 4.75 (P < 0.00001)

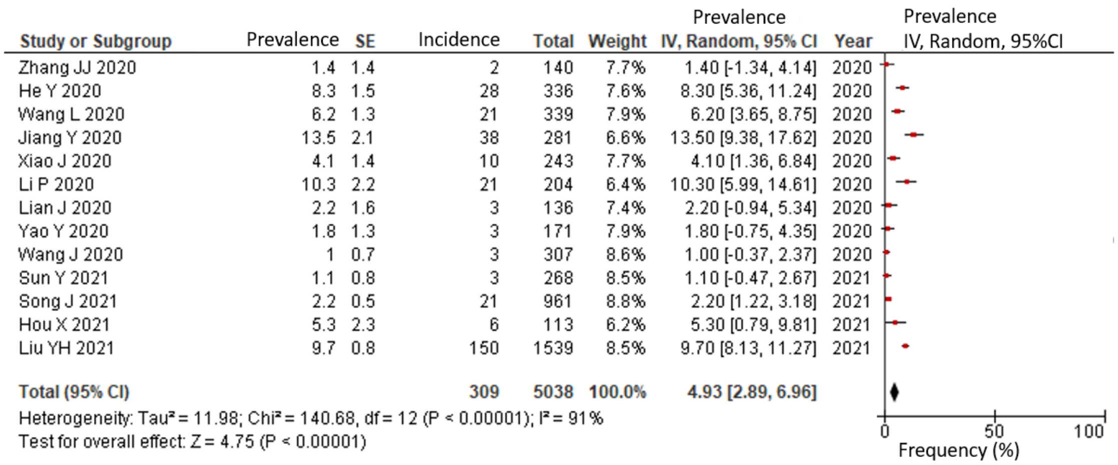

i. Turkey

Study or Subgroup	Prevalence	SE	Incidence	Total	Weight	Prevalence IV, Random, 95% CI	Year	Prevalence IV, Random, 95%CI
Guner R 2020	5.4	1.6	12	222	31.4%	5.40 [2.26, 8.54]	2020	
Degerli E 2021	33.3	6.8	15	45	9.4%	33.30 [19.97, 46.63]	2021	
Turan O 2021	5	0.7	53	1069	35.3%	5.00 [3.63, 6.37]	2021	
Akpinar G 2021	6.8	2.9	6	88	23.9%	6.80 [1.12, 12.48]	2021	
**Total (95% CI)**			**86**	**1424**	**100.0%**	**8.23 [3.47, 12.98]**		

Heterogeneity: Tau² = 16.22; Chi² = 17.39, df = 3 (P = 0.0006); I² = 83%
Test for overall effect: Z = 3.39 (P = 0.0007)

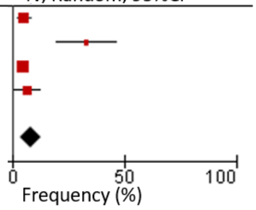

j. India

Study or Subgroup	Prevalence	SE	Incidence	Total	Weight	Prevalence IV, Random, 95% CI	Year	Prevalence IV, Random, 95%CI
Aggarwal A 2020	15.6	6.6	5	32	39.2%	15.60 [2.66, 28.54]	2020	
Goel N 2020	8.6	5.3	3	35	60.8%	8.60 [-1.79, 18.99]	2020	
**Total (95% CI)**			**8**	**67**	**100.0%**	**11.34 [3.24, 19.44]**		

Heterogeneity: Tau² = 0.00; Chi² = 0.68, df = 1 (P = 0.41); I² = 0%
Test for overall effect: Z = 2.75 (P = 0.006)

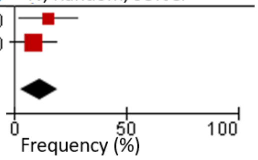

k. Korea

Study or Subgroup	Prevalence	SE	Incidence	Total	Weight	Prevalence IV, Random, 95% CI	Year	Prevalence IV, Random, 95%CI
Yang JM 2020	4.8	0.2	350	7340	50.9%	4.80 [4.41, 5.19]	2020	
Lee SC 2021	3.1	0.3	141	4610	49.1%	3.10 [2.51, 3.69]	2021	
**Total (95% CI)**			**491**	**11950**	**100.0%**	**3.96 [2.30, 5.63]**		

Heterogeneity: Tau² = 1.38; Chi² = 22.23, df = 1 (P < 0.00001); I² = 96%
Test for overall effect: Z = 4.67 (P < 0.00001)

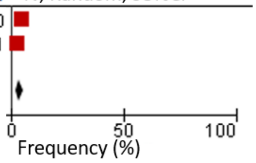

l. Iran

Study or Subgroup	Prevalence	SE	Incidence	Total	Weight	Prevalence IV, Random, 95% CI	Year	Prevalence IV, Random, 95%CI
Tabarsi P 2021	1.2	1.9	1	84	71.0%	1.20 [-2.52, 4.92]	2021	
Mortaz E 2021	10.3	6.2	3	29	29.0%	10.30 [-1.85, 22.45]	2021	
**Total (95% CI)**			**4**	**113**	**100.0%**	**3.84 [-4.25, 11.93]**		

Heterogeneity: Tau² = 20.38; Chi² = 1.97, df = 1 (P = 0.16); I² = 49%
Test for overall effect: Z = 0.93 (P = 0.35)

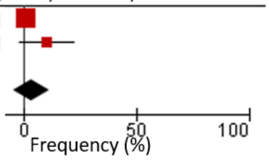

Supplement: S2 Fig — (PDF) [file pone.0276774.s004.pdf]
