# Supplementary figures and images for: Effect of asthma, COPD, and ACO on COVID-19: A systematic review and meta-analysis
Source: PLoS One. 2022 Nov 1;17(11):e0276774. doi: 10.1371/journal.pone.0276774 (PMC9624422; doi:10.1371/journal.pone.0276774)

**S3 Fig. Forrest plots for prevalence of ACO among patients with COVID-19.**

a. USA

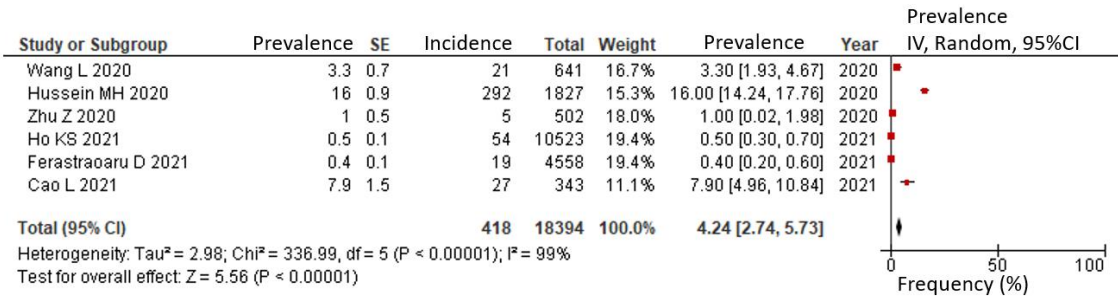

b. UK

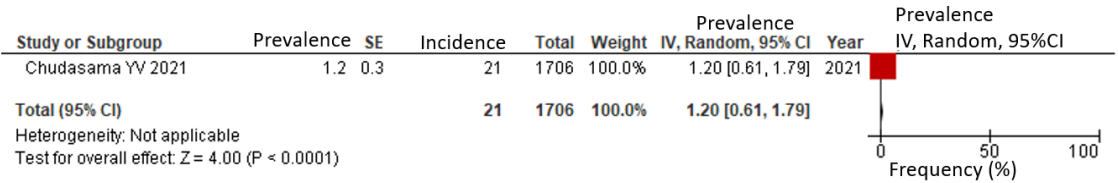

Supplement: S3 Fig — (PDF) [file pone.0276774.s005.pdf]
